# The Black Bird

**Published:** 1912-02

**Authors:** 


### The Black Bird.

(Clipped from a local paper, dated 1865, just after the war.)

"Once upon a midnight dreary, while I pondered weak and weary,
O'er the war of the rebellion and the things that were before—
When I sat absorbed in thinking—brandy cocktails slowly drinking,
Suddenly I saw a blinking, one-eyed figure at my door—
Saw a one-eyed, winking, blinking figure at my chamber door—
Standing there, and nothing more.

Ah! I never shall forget it, how in glancing 'round I met it,
And I ever shall regret it, that I looked toward that door,
For I saw a monstrous figure, like a giant, only bigger,
And there stood a big buck nigger leaning up against my door—
Leaning there, and nothing more.

Straight into the fireplace spying, where my ham and eggs were frying,
I beheld the poker lying near the hearth upon the floor;
Then, with most determined vigor, straight I hurled it at the nigger,
But so quiet was that big nigger that it missed and struck the floor—

Missed the nigger's head completely and fell harmless on the floor—
Struck his heel, and nothing more.

Back into the fireplace looking, where my ham and eggs were cooking,
Shaking, quaking—as no mortal ever shook or quaked before—
I then heard the ugly sinner mutter but these words: 'Some dinner!'
'Twas the only word he'd spoken, 'twas the only word, I'm sure,
When I picked up pluck and answered, 'I shall feed you nevermore!'
This I said, and nothing more.

There I sat, engaged in musing what he meant by thus refusing,
And I then began abusing that big nigger at my door;
'Sure,' said I, 'you must be crazy to be so cursed lazy—
To be so awful lazy as to want to work no more—
Will you ever work for wages—tell me, I implore?'
Quoth the nigger, 'Nevermore!'

'Nigger,' I said, 'horrid demon—nigger still, if slave or freeman—
Think again before you answer this one question, I implore;
Have you yet no sense of feeling—do you mean to live by stealing,
Or by working and fair dealings? Tell me, I implore,
On your honor as a nigger, will you labor as before?'
Quoth the nigger, 'Nevermore!'"

